# Proteomics of Buccal Cavity Mucus in Female Tilapia Fish (*Oreochromis* spp.): A Comparison between Parental and Non-Parental Fish

**DOI:** 10.1371/journal.pone.0018555

**Published:** 2011-04-20

**Authors:** Koe Chun Iq, Alexander Chong Shu-Chien

**Affiliations:** 1 School of Biological Sciences, Universiti Sains Malaysia, Minden, Penang, Malaysia; 2 Assay Development Division, Malaysian Institute of Pharmaceuticals and Nutraceuticals, Ministry of Science, Technology and Innovation, Gelugor, Penang, Malaysia; University of South Florida College of Medicine, United States of America

## Abstract

Mouthbrooding is an elaborate form of parental care displayed by many teleost species. While the direct benefits of mouthbrooding such as protection and transportation of offsprings are known, it is unclear if mouthbrooding offers additional benefits to embryos during incubation. In addition, mouthbrooding could incur negative costs on parental fish, due to limited feeding opportunities. Parental tilapia fish (*Oreochromis* spp.) display an elaborated form of parental care by incubating newly hatched embryos in oral buccal cavity until the complete adsorption of yolk sac. In order to understand the functional aspects of mouthbrooding, we undertake a proteomics approach to compare oral mucus sampled from mouthbrooders and non-mouthbrooders, respectively. Majority of the identified proteins have also been previously identified in other biological fluids or mucus-rich organs in different organisms. We also showed the upregulation of 22 proteins and down regulation of 3 proteins in mucus collected from mouthbrooders. Anterior gradient protein, hemoglobin beta-A chain and alpha-2 globin levels were lower in mouthbrooder samples. Mouthbrooder oral mucus collectively showed increase levels of proteins related to cytoskeletal properties, glycolytic pathway and mediation of oxidative stress. Overall the findings suggest cellular stress response, probably to support production of mucus during mouthbrooding phase.

## Introduction

Parental care is described as a post-fertilization parental behavior to increase offspring survival and fitness [1]. The major benefits of parental care can be broadly divided into offspring protection and embryonic development [2]. Consequently, parental care activities could incur negative costs on individual parent, since increase in energy expenditure during brood care activities coupled with reduction in feeding opportunities may eventually result in decline of endogenous energy reserves [1]. Over 20% of teleost families are known to exhibit parental care behaviours [3]. Among them, members of the Cichlidae family exhibit diversified patterns of parental care, which include egg guarding and mouthbrooding activities [2]. Mouthbrooding, the incubation of offspring in the parental mouth, is displayed by at least 9 families of teleost fish [4]. The tilapia fish (*Oreochromis* spp) are uniparental mouthbrooders, with the females incubating newly fertilized eggs and larvae in the mouth cavity, usually until the complete absorption of larva yolk sac [5]. Mouthbrooding undoubtedly offers the benefit of physical protection from predators or environmental stressors and the capacity to transport fries to a more conducive environment [6]. Similar to pouch-bearing and viviparous species, mouthbrooding may allow embryos to develop to a more advanced and less susceptible stage [7], [8]. Tilapia offsprings raised from mouthbrooding possessed higher rate of protection from ectoparasite as compared to those raised through artificial incubation, indicating the possibility of passive immunity transfer during mouthbrooding [9], [10]. The detection of the yolk protein precursor vitellogenin in mouthbrooding tilapia surface and oral mucus seems to suggest maternal-embryo nutrient transfer [11]. In comparison, known negative consequences of mouthbrooding include starvation, increased energy expenditure, hypoxia, decrease in immune function, limited locomotion and reduced reproductive success [8], [12], [13].

Fish mucus is involved in an array of biological activities including mechanical protection, anti-infection, respiration, communication, nest building and parental care [14]. In relation to mouthbrooding, parental oral mucus secretion may facilitate lubrication, trapping of food particles, provide pathogenic defence and buffering of pH for digestion [15], [16]. However, the direct benefits of parental oral mucus towards offspring during mouthbrooding remain to be elucidated. Adaptations at physiological and biochemical levels to enable manipulation of oral mucus composition and quantity during mouthbrooding have been reported [17]. In tilapia (*O. mossambica*), there are mouthbrooding-related variations in concentration of various oral mucosal substances, including mucins and glycoproteins [17]. In addition, we previously demonstrated the occurrence of biochemical changes in the epidermal mucus of parental discus fish during parental care phase, and deduced that these changes could possibily be crucial to larval development and protection of parental fish [18], [19]. Insights on the protein composition of oral mucus of mouthbrooders in relation to mouthbrooding activities may provide useful knowledge on the functional aspects of this behavior. Proteomics approach have been widely used to profile proteome of mucus samples from various sources, including oral, olfactory cleft [20], nasal [21], cervical [22] and brancheolar tissue [23]. The aim of this present study is to compare the proteome of tilapia buccal cavity mucus during parental-care and non-parental care phase.

## Methods

### Fish Husbandry and Selection of Mouthbrooders

Sexually mature red tilapias at ratio of 4 females to 1 male were raised in 200 L raceway tanks equipped with flow-through fresh water at temperature of 30°C under natural photoperiod. Fish were fed with commercial pellet twice daily at 0900 and 1600 hrs. In order to identify individual fish displaying mouthbrooding activities, daily observation was carried out during feeding time. Individuals displaying signs of mouthbrooding such as territorial behavior and non-feeding activity were isolated. These mouthbrooders were kept until the day of mucus collection, as described below.

### Oral Mucus Collection and Sample Preparation

Oral mucus was sampled from 6 female fish randomly chosen from the raceway tanks population and designated as non-mouthbrooder mucus samples. As for mouthbrooders, oral mucus sampling was done on the 8^th^–10^th^ day of mouthbrooding. Mucus was collected from surface of the buccal cavity region using glass pipettes and transferred into microtubes at 4°C, followed by centrifugation at 13,200 rpm at 4°C for 20 minutes. Pre-chilled acetone was added into the supernatant at ratio of 4 acetone∶1 sample (v/v). Mixture was then incubated at −20°C for 2 hours, followed by centrifugation at 15,000 g, 4°C for 10 minutes. The resulting pellet was dissolved in rehydration buffer [8 M urea, 50 mM DTT, 4% CHAPS, 0.2% ampholyte 3/10 (Bio-Rad, Hercules, CA, USA), 0.0002% bromophenol blue and deionized distilled water].

### 2-D Gel Electrophoresis and Gel Analysis

Protein concentration was determined using RC DC protein assay kit (Bio-Rad, Hercules, CA, USA). Analytical gels were prepared by passively rehydrating 17 cm pH 3–10 NL ReadyStrip IPG strips (Bio-Rad, Hercules, CA, USA) in 300 µL of rehydration buffer containing 60 µg of protein for 16 hours. For gels used for mass spectrometry, 3 mg of protein in 300 µL of rehydration buffer was applied. Isoelectric focusing (IEF) was carried out using PROTEAN IEF cell (Bio-Rad, Hercules, CA, USA) at 250 V for 20 minutes, followed by 10,000 V (2.5 hours) and 10,000 V, 40,000 Vhr (4 hours). Following IEF separation, IPG strips were equilibrated with the first equilibration solution [6 M urea, 0.05 M Tris-HCl (pH 8.8), 2% SDS, 20% glycerol, 2% (w/v) DTT] for 15 minutes with gentle shaking. This was followed by another equilibration with a second equilibration solution [6 M urea, 0.05 M Tris-HCl (pH 8.8), 2% SDS, 20% glycerol, 2.5% (w/v) iodoacetamide] solution for 15 minutes with gentle shaking. Equilibrated strips were applied onto 15% SDS-PAGE gel for the second dimension separation using PROTEAN II XL vertical electrophoresis system (Bio-Rad, Hercules, CA, USA) at constant ampere of 16 A per gel for 30 minutes before increasing to 24 A per gel until the end of the electrophoresis run. Precision Plus Protein standard (Bio-Rad, Hercules, CA, USA) was used as molecular weight marker. Analytical gels were stained using the Vorum silver staining method. Briefly, gels were immersed in fixing solution (50% methanol, 12% acetic acid, 0.05% formalin) overnight and staining solution (0.2% silver nitrate, 0.076% formalin) for 20 minutes. Stained gels were washed twice in deionized distilled water for 1 minute followed by immersion in a developing solution (6% sodium carbonate, 0.05% formalin, 0.0004% sodium thiosulfate) and before termination in a stopping solution (50% methanol, 12% acetic acid) for 5 minutes. Gels used for mass spectrometry were stained with Coomassie Brilliant Blue (CBB). Briefly, gels were fixed in 50% methanol and 10% acetic acid solution for 2 hours. Fixed gels were then stained in staining solution [0.1% (w/v) Coomassie Brilliant blue R-250, 10% acetic acid] for 4 hours. Destaining was in 10% acetic acid.

Silver stained gels were scanned using GS-800 calibrated densitometer (Bio-Rad) and analyzed using PDQuest version 7.3.1 (Bio-Rad, Hercules, CA, USA). A single analytical gel was prepared from each mucus sample, amounting to 6 mouthbrooder and non-mouthbrooder replicate analytical gels respectively. All gels were scanned using the GS-800 densitometer (Bio-Rad, Hercules, CA, USA) and protein spots were analyzed using PDQuest version 7.3.1 (Bio-Rad, Hercules, CA, USA). Gels were analyzed for spot detection, background subtraction and protein spot OD intensity quantification using the 3D imaging function in the software to eliminate artifact spots. One non-mouthbrooder replicate gel was selected as the master gel, for purpose of automatic alignment and spot matching with other gels. For comparison of mouthbrooder and non-mouthbrooder proteomes, two-tailed t-test (p<0.05) analysis of mean spot intensities was carried out.

### In-gel Digestion and Zip Tip Desalting

For mass spectrometry analysis, spots of interest were excised from CBB gels using new scalpel blades and transferred to 200 µL microtubes. Gel pieces were coarsely grounded up using new pipette tips, destained 3 times with 100 µL of 50 mM ammonium bicarbonate/50% acetonitrile (v/v) for 5 minutes and subsequently dehydrated 3 times with 50 µL acetonitrile for 5 minutes. Then, gel pieces were thoroughly dried using a vacuum centrifuge followed by rehydration with 15 µL of digestion solution (12.5 ng/µL trypsin in 50 mM ammonium bicarbonate solution) at 4°C for 30 minutes. Gel pieces were then incubated overnight in 15 µL of 50 mM ammonium at 37°C. After incubation, gel pieces were allowed to cool to room temperature followed by centrifugation at 6000 rpm for 10 minutes. The resulting supernatant was removed and stored. Leftover pellet was resuspended in 15 µL of 20 mM ammonium bicarbonate, followed by centrifugation at 6000 rpm for 10 minutes. The supernatant was then removed and pooled with earlier samples. Resulting pellet was treated with 15 µL of 5% formic acid in 50% aqueous acetonitrile for 10 minutes, followed by centrifugation at 6000 rpm for 10 minutes. Supernatant was collected and pooled with the previous mixtures.

Pooled extract mixtures were dried thoroughly using vacuum centrifuge. Dried extract was re-dissolved in 10 µL of 0.5% formic acid and subsequently desalted using ZipTip C18 (Millipore, Bedford, MA, USA). Briefly, Ziptip was filled with acetonitrile and washed with deionized distilled water. Extract solution was pipetted in and out at least 10 times with ZipTip to ensure the proper retention of peptides before desalting with 0.5% formic acid. Peptides were then extracted with 0.5% formic acid in 1∶1 (v/v) water∶acetonitrile and vacuum dried.

### Mass Spectrometric Analysis and Protein Identification

Peptides were re-dissolved in 1 µL of matrix solution consisting 5 mg/ml of a-cyano-4-hydroxycinnamic acid in 0.1% TFA, 50% ACN in MilliQ water. Peptide mixture was spotted onto the MALDI target plate, allowed to dry prior to mass spectrometry analysis. Mass spectrometry was performed using the 4800 MALDI-TOF/TOF Analyzer (Applied Biosystems, Framingham, Massachusetts) using settings and parameters described earlier [18]. MS-MS/MS data was interpreted using Data Explorer version 4.9 (Applied Biosystem). Peptide sequences were obtained by calculating the differences residue mass between the adjacent fragment ion peaks. MS/MS sequences were subjected to different protein database searching tools such as from NCBI, PROSITE, and Pfam to identify possible matches.

### Ethics statement

All procedures involving animal handling in this study complied with the Ethics Guidelines as formulated by the Animal Ethics Committee, Universiti Sains Malaysia and was approved under the registration number of USM/Animal Ethics Approval/2010/(62)(250).

## Results and Discussion

**Figure 1:**
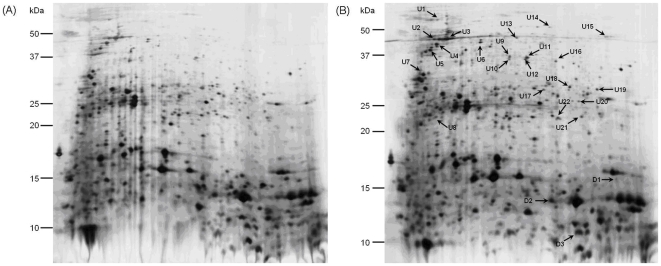
2-D gel maps of tilapia fish buccal cavity mucus. (A) Gel map for non-parental mucus proteins. (B). Gel map for parental mucus proteins. A total of 60 µg of proteins (n = 6) were separated by 2-DE using 17 cm, pH 3–10 NL IPG strip and 15% SDS-PAGE. The 2-D gels were stained using Vorum silver staining and scanned by GS-800 calibrated densitometer (Bio-Rad) and protein spots were analyzed using PDQuest version 7.3.1 (Bio-Rad).

**Figure 2:**
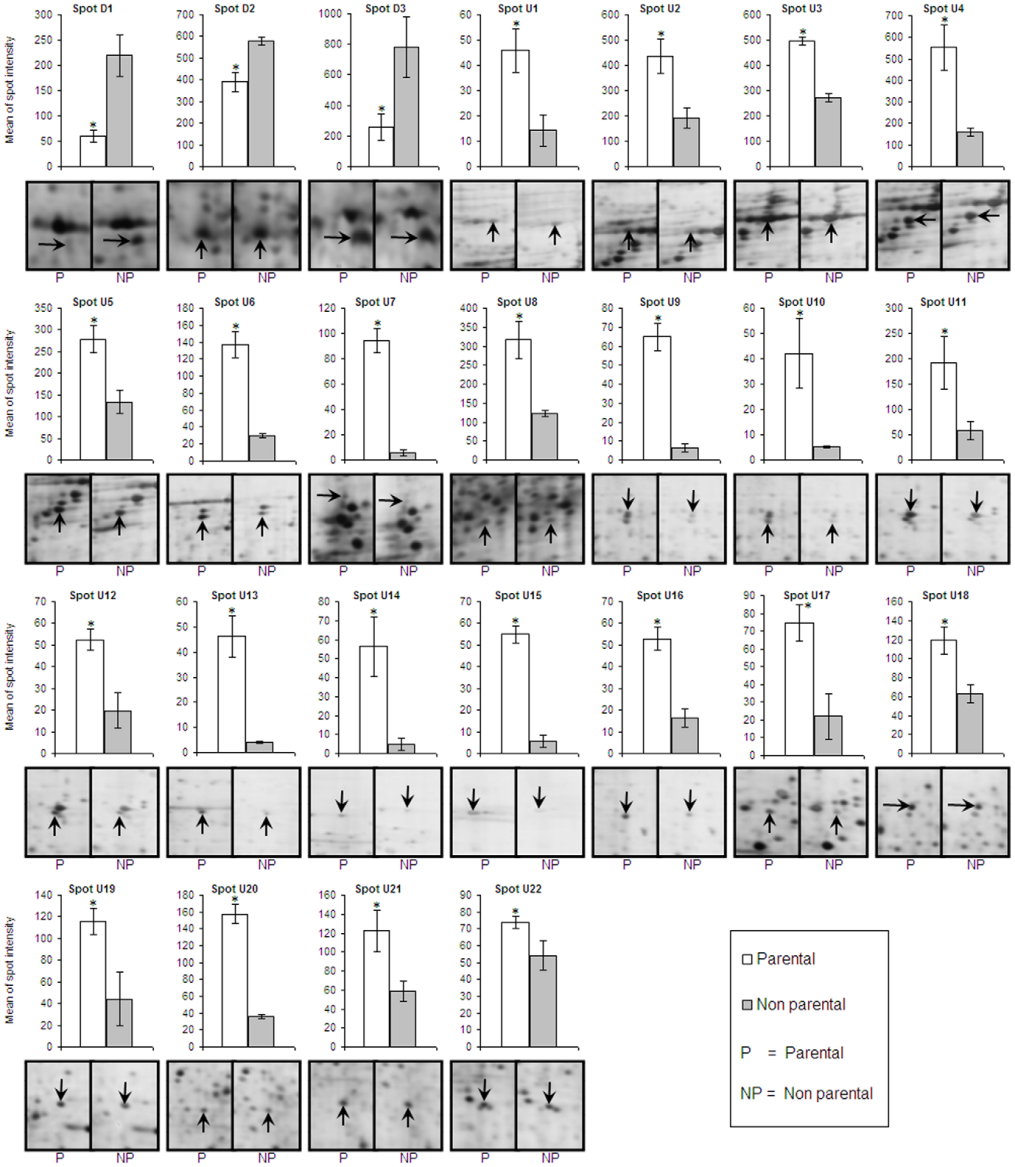
Significant difference between the regulation of tilapia fish parental and non-parental (n = 6) buccal cavity mucus proteins based on two-tailed *t*-test (*p<0.05). Bars represent the mean ± SEM of spot intensities.

Figure 1 shows representative of the silver stained mouthbrooder oral mucus and non-mouthbrooder oral mucus 2-DE gels. An average of 320 and 317 spots were detected in mouthbrooder and non-mouthbrooder mucus 2-DE gels, respectively. A total of 90 spots were found to fit the criteria described in spot analysis and were excised for mass spectrometry analysis. Using two-tailed *t*-test (P<0.05) to compare mean of spot intensity between mouthbrooder and non-mouth brooder, (n = 6), we identified 22 proteins with significantly higher expression in mouthbrooder mucus, while 3 proteins showed lower expression (Figure 2). Lists of down-regulated and up-regulated proteins together with the corresponding mass spectrometry characteristics are shown in Table 1, Table 2 and Table S1 respectively.

**Table 1:** List of down-regulated proteins in tilapia fish parental buccal cavity mucus identified using MALDI-TOF/TOF and MASCOT.

Spot	Accession number	Protein	Organism	MW (kDa) Exp./Theo.^a^	p*I* Exp./Theo.^b^
D1	ABB96969	anterior gradient-2-like protein 2	*Salmo salar*	15.6/19.6	8.72/8.91
D2	AAY79276	hemoglobin beta-A chain	*Siniperca chuatsi*	12.9/16.0	7.32/7.82
D3	ABF67513	alpha-2 globin	*Sparus aurata*	10.3/15.9	7.93/8.79

a) Experimental and theoretical molecular weight (kDa).

b) Experimental and theoretical p*I*.

**Table 2:** List of up-regulated proteins in tilapia fish parental buccal cavity mucus identified using MALDI-TOF/TOF and MASCOT.

Spot	Accession number	Protein	Organism	MW (kDa) Exp./Theo.^a^	p*I* Exp./Theo.^b^
U1	AY559446	alpha-1-antitrypsin	*Oreochromis mossambicus*	59.4/23.5	4.79/5.51
U2^c^	O42161	actin,cytoplasmic 1 (beta actin)	*Salmo salar*	42.2/42.1	4.85/5.30
U3^c^	AJ537421	beta actin	*Dicentrarchus labrax*	42.5/42.1	5.00/5.29
U4^c^	AAY52024	beta actin	*Gasterosteus aculeatusi*	38.4/40.4	4.78/5.56
U5^c^	AAY52025	beta actin	*Pungitius pungitius*	34.1/40.5	4.64/5.29
U6^c^	AAG17453	beta actin	*Rhodeus notatus*	40.0/42.0	5.78/5.38
U7^c^	AAQ21403	beta actin	*Monopterus albus*	28.4/42.1	4.42/5.31
U8^c^	AAK83921	actin	*Fundulus heteroclitus*	22.5/16.0	4.76/5.93
U9	AAD23573	glyceraldehyde-3-phosphate dehydrogenase	*Astatotilapia burtoni*	30.0/36.2	6.42/6.40
U10	AAD23573	glyceraldehyde-3-phosphate dehydrogenase	*Astatotilapia burtoni*	29.7/36.2	6.14/6.40
U11^c^	AAD23573	glyceraldehyde-3-phosphate dehydrogenase	*Astatotilapia burtoni*	29.9/36.2	6.80/6.40
U12	AAD23573	glyceraldehyde-3-phosphate dehydrogenase	*Astatotilapia burtoni*	29.7/36.2	6.76/6.40
U13	BAD17943	Phosphoglycerate kinase	*Potamotrygon motoro*	43.3/42.1	6.58/7.05
U14	AAH92869	enolase 3, (beta, muscle)	*Danio rerio*	52.2/47.8	7.28/6.20
U15^c^	CAD38126	Cytokeratin type IIE	*Acipenser baerii*	44.2/51.4	8.49/5.06
U16	ABN80442	lactate dehydrogenase B	*Poecilia reticulata*	29.5/28.7	7.42/7.74
U17	ABB17040	heat shock cognate 70	*Fundulus heteroclitus macrolepidotus*	26.4/71.1	7.15/5.27
U18	NP_999862	proteasome (prosome, macropain) subunit, alpha type, 4	*Danio rerio*	26.7/29.6	7.72/7.57
U19	NP_001098270	heat shock protein 70 cognate	*Oryzias latipes*	26.4/76.6	8.31/5.80
U20	ABN80450	triose phosphate isomerase B	*Poecilia reticulata*	25.3/26.9	7.91/6.90
U21	CAG12406	unnamed protein product	*Tetraodon nigroviridis*	22.5/21.5	7.97/7.66
U22	ABF01135	natural killer enhancing factor	*Scophthalmus maximus*	22.7/22.1	7.31/5.58

a) Experimental and theoretical molecular weight.

b) Experimental and theoretical p*I*.

c) Taxonomy filter on MASCOT was applied using Actinopterygii (ray-finned fishes).

One protein showing lower expression in parental oral mucus sample is the Anterior gradient 2 protein (AGR2), which was first identified in embryonic *Xenopus laevis* cement gland, a mucus-secreting anterior organ [24]. Elsewhere, transcripts of AGR2 have been detected in mammalian mucus-rich organs such as lung, trachea and the digestive organs [25]. In zebrafish, AGR2 mRNA is expressed in mucus secreting cells located in ectoderm and endoderm derived organs [26]. AGR2 mRNA was also localized in epithelial layers of gill and intestine of Atlantic salmon [27]. AGR2 also belongs to a family of endoplasmic reticulum proteins that facilitate folding of proteins involved in the secretory pathway [28]. Transcriptome studies on responses towards infection have reported elevated AGR2 expression in salmon gills infected by amoebic gill diseases, while in mycobacterium infected-zebrafish, its expression was downregulated [27], [29]. The regulation of AGR2 by the hormone estrogen has been reported previously [30], [31]. Mouthbrooding black-chinned tilapia (*Sarotherodon melanotheron*) posses lower androgen and estradiol levels as compared to non mouthbrooding fish [32]. Therefore, the reduction of AGR2 level in our tilapia mouthbrooder mucus could be due to lower levels of estrogen during mouth brooding phase. Two other proteins, identified as hemoglobin beta-A chain and alpha-2 globin, respectively, were also downregulated in mouthbrooder mucus. Both these proteins were earlier reported in epidermal mucus from other types of biological fluid [20], [21].

Expression of an anti-trypsin protein was upregulated in mouthbrooder oral mucus. Elsewhere, inhibitors of various proteases such as serpins, α-2 macroglobulin and cysteine have been isolated from teleost epidermal tissues and mucus [33], [34], [35]. Functionally, these inhibitors protect the host from undesired intracellular and external proteolytic activities, defense against pathogens and regulate intracellular proteolysis activities associated with a diverse set of biochemical pathways [36]. The upregulation of trypsin-like inhibitor in mouthbrooder oral mucus could be important in protection of both larvae and mouthbrooding parent fish from pathogen invasion. In human nasal fluid, the upregulation of alpha anti trypsin is reported to protect tissues from degradatory action of proteolytic enzymes during allergen-triggered inflammation [37]. Parental discus fish, also showed unique expression of C-lectin, a carbohydrate binding protein with anti microbial properties in epidermal mucus during parental-care phase [18].

We also detected upregulated levels of the cytoskeletal beta-actins in mouthbrooder mucus. Cytoskeletal proteins have been reported in nasal mucus [20]. In nasal epithelial and airway goblet cells, actin filaments have been shown to regulate mucin secretion [38], [39]. Actin was also detected in Atlantic salmon mucus [40]. In airway goblet cells, actin filaments interact with secretory granules to mediate their movements to the cellular apical membrane for mucus secretion [41]. Upregulated expression of an actin capping protein was also reported in mucus of discus fish during parental care period [18].

In tandem, the elevated expression of a type II cytokeratin in mouthbrooder mucus sample could also to be linked with the epithelial cell cytoskeletal machinery. Cytokeratins are also known sensitive markers of stressed-induced epithelial cells cytoskeletal differentiation [42]. Increased level of keratins was also reported in epidermal mucus secretion of salmon infected with sea lice [40]. In rainbow trout, a type II cytokeratin found in epidermal mucus displayed pore-forming properties for antibacterial purpose [43]. In hagfish, epidermal cells synthesize and secrete homologues of cytokeratin II proteins as biopolymers to regulate the viscoelastic and cohesive properties of body mucus [44].

The elevated levels of several enzymes belonging to the glycolytic pathways in parental oral mucus (glyceraldehyde-3-phosphate dehydrogenase (GAPDH), phosphoglycerate kinase, enolase 3, lactate dehydrogenase B, fructose-biphosphate aldolase C, and triose phosphate isomerase B), indicate higher cellular metabolic activities, possibly to support intensified epithelial cell proliferation and mucus production during mouthbrooding. The elevation of several glycolytic enzymes in mucus was also reported in parental discus fish [18]. Increased glycolysis could also function to counteract hypoxia conditions during mouthbrooding activities [45]. Glycolytic enzymes have also been detected in several types of biological fluids including oral mucus, saliva and cervical fluid [20], [46].

Proteasomes are essential regulators of cell-cycle, signal transduction, immunity and chaperon activities through their intracellular proteins degradation activities. We detected an upregulated expression of the proteasome subunit alpha type 4 in parental oral mucus. In human epithelial cells, mucus production is triggered by tumor necrosis factor activating several intracellular signal transduction cascades [47]. Among them, the nuclear factor-κB pathway requires proteasome-mediated degradation of phosphorylated complexes to eventually release the nuclear factor-κB complex, which acts as a transcription factor activating numerous genes vital for mucus production [48]. Speculatively, proteasome could be important for mucus production, immune response, DNA repair, metabolism, apoptosis, chaperoning and cell cycle progression of mucosal or buccal cavity cells during oral incubation.

NK cell enhancing factor (NKEF) has a wide range of expression and belongs to a new class of the peroxiredoxin gene family found in diverse organisms. In several teleost species, upregulation expression of NKEF is linked to pathogenic infection [49], [50]. Characterization of NKEF has shown that this protein plays a role in antioxidation, immunity and cellular proliferation [51]. Therefore, the higher expression of NKEF proteins in mouthbrooder oral mucus could indicate increased antioxidant and immunity oral epithelial cells during parental care phase. The presence of several peroxiredoxin isoforms were also reported in human oral cleft mucus and are speculated to have antioxidant defense function in oral epithelial cells [20]. Elevated expression of thioredoxin peroxireductase, which belongs to the peroxiredoxin family was also detected in mucus secreted by discus fish during parental care [18].

Another protein involved in stress mediation, the heat shock protein (HSP) 70 kDa was also upregulated in parental oral mucus. Cellular HSPs are produced to respond to a wide range of stress such as heat shock, mechanical stress, infections, oxidants and cytokines-related induction [52]. In mammals and teleost, different HSP isoforms have been reported to show increased expression in skin tissues under stressful conditions [53], [54]. HSP have been reported in mucosal defense mechanism of rat intestinal tissue [55]. Mucus secretory cells in lungs of rats expressed higher levels of HSP under presence of cigarette contaminants [56]. In mucus of human oral cleft, several members of the HSP family contribute to 11% of total overall identified proteins, which signify the importance of HSPs for protection of the epithelial layer [20].

Proteins such as the glycolysis enzymes, HSP and keratins have been highlighted as proteins that are repeatedly identified from studies employing 2 dimensional electrophoresis (2-DE) technique on both human and rat tissues [57]. Although this occurrence could be due to the limitations of the 2-DE platform, it has also been suggested that these proteins could collectively represent a group of common cellular sensors [57], [58]. The identification of these proteins and their upregulation in mouthbrooder oral mucus imply a stress response during mouthbrooding phase, which could be due to hyperplasia and desquamation of the oral epithelial layer.

## Supporting Information

Table S1 **Mass spectrometry details (PMF and MS/MS) of identified upregulated and down regulated proteins in parental tilapia buccal cavity mucus.**
(DOCX)
