# Supplementary material for: Proteomics of Buccal Cavity Mucus in Female Tilapia Fish (Oreochromis spp.): A Comparison between Parental and Non-Parental Fish
Source: PLoS One. 2011 Apr 20;6(4):e18555. doi: 10.1371/journal.pone.0018555 (PMC3080365; doi:10.1371/journal.pone.0018555)
Supplement: Table S1 — Mass spectrometry details (PMF and MS/MS) of identified upregulated and down regulated proteins in parental tilapia buccal cavity mucus. (DOCX) [file pone.0018555.s001.docx]

Table S1. Mass spectrometry details (PMF and MS/MS) of identified upregulated and down regulated proteins in parental tilapia buccal cavity mucus

|  |  |  | PMF | | | MS/MS | |
| --- | --- | --- | --- | --- | --- | --- | --- |
| Spot | Protein | Organism | Score^a^ | No. of peptides matched | Sequence coverage | Score^b^ | Sequence of closest matching peptides |
| D1 | anterior gradient-2-like protein 2 | *Salmo*  *Salar* | 75 | 4 | 35% | 49 | 113-HLSPDGQYVPR-123  124-IIFVDPSMTVR-134 |
| D2 | hemoglobin beta-A chain | *Siniperca chuatsi* | 104 | 3 | 23% | 85 | 97-LHVDPDNFR-105  119-FGPSVFTADAQEAWQK-134 |
| D3 | alpha-2 globin | *Sparus*  *aurata* | 110 | 2 | 18% | 92 | 75-IDDLTGALSSLSELHAFK-92  93-LRVDPANFR-101 |
| U1 | alpha-1-antitrypsin | *Oreochromis mossambicus* | 218 | 3 | 26% | 173 | 76-SGFSPLEEFLNDIK-89  90-QYYSGEIFQVDFTKPEEAAAEINK-113  129-DLDPEMAMVLINYVYFR-145 Oxidation (M) |
| U2^c^ | actin,  cytoplasmic 1  (beta actin) | *Salmo salar* | 462 | 8 | 40% | 404 | 85-IWHHTFYNELR-95  148-TTGIVMDSGDGVTHTVPIYEGYALPHAILR-177  239-SYELPDGQVITIGNER-254  292-DLYANTVLSGGTTMYPGIADR-312 |
| U3^c^ | beta actin | *Dicentrarchus*  *labrax* | 461 | 9 | 45% | 402 | 85-IWHHTFYNELR-95  148-TTGIVMDSGDGVTHTVPIYEGYALPHAILR-177  239-SYELPDGQVITIGNER-254  292-DLYANTVLSGGTTMYPGIADR-312 |
| U4^c^ | beta actin | *Gasterosteus*  *aculeatusi* | 398 | 7 | 40% | 339 | 78-IWHHTFYNELR-88  89-VAPEEHPVLLTEAPLNPK-106  232-SYELPDGQVITIGNER-247  285-DLYANTVLSGGTTMYPGIADR-305 |
| U5^c^ | beta actin | *Pungitius*  *pungitius* | 514 | 8 | 46% | 442 | 78-IWHHTFYNELR-88  89-VAPEEHPVLLTEAPLNPK-106  141-TTGIVMDSGDGVTHTVPIYEGYALPHAILR-170 Oxidation (M)  232-SYELPDGQVITIGNER-247  284-DLYANTVLSGGTTMYPGIADR-305 |
| U6^c^ | beta actin | *Rhodeus*  *notatus* | 109 | 10 | 35% | 37 | 96-VAPEEHPVLLTEAPLNPK-113  239-SYELPDGQVITIGNER-254 |
| U7^c^ | beta actin | *Monopterus*  *albus* | 152 | 9 | 39% | 77 | 29-AVFPSIVGRPR-39  85-IWHHTFYNELR-95  96-VAPEEHPVLLTEAPLNPK-113  239-SYELPDGQVITIGNER-254 |
| U8^c^ | actin | *Fundulus*  *heteroclitus* | 112 | 6 | 49% | 54 | 16-AVFPSIVGRPR-26  83-VAPEEHPVLLTEAPLNPK-100 |
| U9 | glyceraldehyde  -3-phosphate dehydrogenase | *Astatotilapia*  *burtoni* | 111 | 2 | 8% | 98 | 310-LISWYDNEYGYSNR-323 |
| U10 | glyceraldehyde  -3-phosphate dehydrogenase | *Astatotilapia*  *burtoni* | 241 | 7 | 25% | 184 | 235-VPVADVSVVDLTCR-248  310-LISWYDNEYGYSNR-323 |
| U11^c^ | glyceraldehyde  -3-phosphate dehydrogenase | *Astatotilapia*  *burtoni* | 106 | 8 | 35% | 47 | 93-YVVESTGVFLSVEK-106  163-VIHDSFGIEEALMTTVHAYTATQK-186  310-LISWYDNEYGYSNR-323 |
| U12 | glyceraldehyde  -3-phosphate dehydrogenase | *Astatotilapia*  *burtoni* | 176 | 8 | 34% | 118 | 235-VPVADVSVVDLTCR-248  310-LISWYDNEYGYSNR-323 |
| U13 | phosphoglycerate  kinase | *Potamotrygon*  *motoro* | 101 | 4 | 15% | 87 | 14-AAIPSIQHCLDHGAK-28  129-LGDVYVNDAFGTAHR-143 |
| U14 | enolase 3,  (beta, muscle) | *Danio*  *rerio* | 212 | 7 | 26% | 179 | 33-AAVPSGASTGVHEALELR-50  203-DATNVGDEGGFAPNILENNEALELLK-228 |
| U15^c^ | Cytokeratin type IIE | *Acipenser*  *baerii* | 71 | 8 | 21% | 38 | 370-LALDIEIATYR-380 |
| U16 | lactate  dehydrogenase B | *Poecilia*  *reticulata* | 267 | 5 | 37% | 234 | 24-VTVVGVGQVGMACAVSILLR-43  44-DLCDELALVDVMEDR-58  92-LVVVTAGVR-100  179-LGIHASSFNGWVLGEHGDTSVPVWSGANVAGVNLQK-214 |
| U17 | heat shock  cognate 70 | *Fundulus*  *Heteroclitus*  *macrolepidotus* | 327 | 14 | 31% | 232 | 4-GPAVGIDLGTTYSCVGVFQHGK-25  26-VEIIANDQGNR-36  37-TTPSYVAFTDTER-49  138-TVNNAVITVPAYFNDSQR-155  160-DAGTISGLNVLR-171  221-STAGDTHLGGEDFDNR-236 |
| U18 | proteasome  (prosome,  macropain)  subunit, alpha  type, 4 | *Danio*  *rerio* | 367 | 6 | 37% | 321 | 9-TTIFSPEGR-17  55-LLDEVFFSEK-64  68-LNEDMACSVAGITSDANVLTNELR-91  97-YLLQYQEPIPCEQLVTALCDIK-118  128-RPFGVSLLYMGWDK-141 Oxidation (M)  142-HYGFQLYQSDPSGNYGGWK-160 |
| U19 | heat shock protein 70 cognate | *Oryzias*  *latipes* | 319 | 12 | 25% | 226 | 4-GPAVGIDLGTTYSCVGVFQHGK-25  37-TTPSYVAFTDTER-49  138-TVNNAVITVPAYFNDSQR-155  160-DAGTISGLNVLR-171  221-STAGDTHLGGEDFDNR-236 |
| U20 | triose phosphate isomerase B | *Poecilia*  *reticulata* | 428 | 10 | 51% | 321 | 5-FFVGGNWK-12  58-FGVAAQNCYK-67  84-DCGVNWVILGHSER-97  99-HVFGESDELIGQK-111  159-VVLAYEPVWAIGTGK-173  193-TNVSEAVANSVR-204  224-DVDGFLVGGASLKPEFIDIINAK-246 |
| U21 | unnamed protein  product | *Tetraodon*  *nigroviridis* | 156 | 5 | 27% | 102 | 10-IIFVVGGPGSGK-21  32-YGYTHLSSGDLLR-44  89-GFLIDGYPR-97  156-ATEPVIAFYESR-167 |
| U22 | natural killer  enhancing factor | *Scophthalmus*  *maximus* | 88 | 2 | 10% | 74 | 8-IGMPAPDFK-16 Oxidation (M)  140-QITINDLPVGR-150 |

a) PMF score is -10*log(*P*), where *P* is the probability that the observed match is random event, it is based on NCBInr database using MASCOT searching program as MALDI-TOF/TOF data. Proteins scores are significant when *p*< 0.05.

b) MS/MS score is -10*log(*P*), where *P* is the probability that the observed match is a random event, it is based on NCBInr database using MASCOT searching program as MALDI-TOF/TOF data. Protein scores are significant when *p*< 0.05.

c) Taxonomy filter on MASCOT was applied using Actinopterygii (ray-finned fishes).
